# Update on: high but slightly declining COVID-19 vaccine acceptance and reasons for vaccine acceptance, Finland April to December 2020

**DOI:** 10.1017/S0950268821001680

**Published:** 2021-08-03

**Authors:** Charlotte C. Hammer, Veronica Cristea, Timothee Dub, Jonas Sivelä

**Affiliations:** 1 Department of Health Security, Finnish Institute for Health and Welfare (THL), Helsinki, Finland; 2 European Programme for Intervention Epidemiology Training (EPIET), European Centre for Disease Prevention and Control (ECDC), Stockholm, Sweden

**Keywords:** COVID-19, KAP study, vaccine acceptance, vaccine hesitancy

## Abstract

We update our previous insights into COVID-19 vaccine acceptance and hesitancy in Finland. Vaccine acceptance increased from 64% (November/December 2020) to 74% (April 2021). However, there was a group of participants that were preferring to wait to get vaccinated ranging from 6% of over-64-years-olds to 29% of under-30-years-olds. The previously identified enablers convenience (below-50-years-olds), worry about severe disease and protection for oneself (above-50-years-olds) were no longer significantly associated with increased vaccine acceptance. Understanding barriers and enablers behind vaccine acceptance is decisive in ensuring a successful implementation of COVID-19 vaccination programs, which will be key to ending the pandemic.

In our recent publication [1], we reported on the results of the first four rounds of data collection of the COSMO study in Finland. Since then, we conducted another round of data collection between 16 April and 19 April 2021^1^.

In November/December 2020, vaccine acceptance was 64%, the lowest since the beginning of data collection in April 2020. However, in April 2021, vaccine acceptance had increased to 74%, the highest ever recorded since the beginning of data collection. The percentage of participants strongly agreeing with accepting a vaccine increased from 37% in November/December 2020 to 50% in April 2021. The clear drop-off in vaccine acceptance previously described at age 50 was replaced with a more gradual age gradient (Fig. 1).

**Fig. 1.**
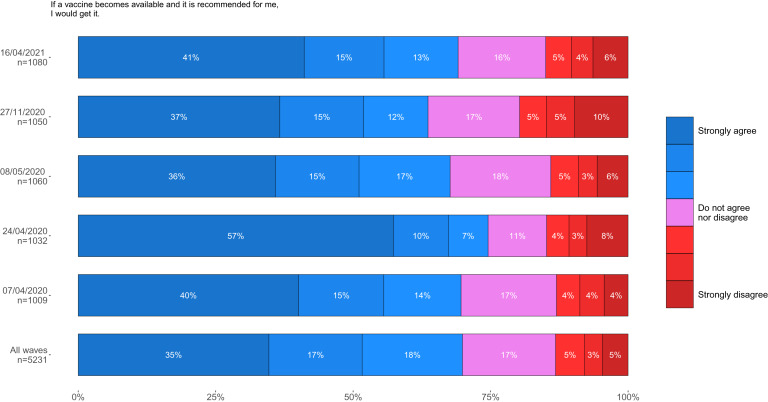
Self-declared likelihood of accepting a COVID vaccine if/when one is offered during the fifth round (April 2021) by age group.

While considering a recommendation from a healthcare provider remained associated with a higher likelihood to accept vaccination in the age group below 50 (estimate: 0.16, 95% CI 0.01–0.32, *P*-value = 0.041), convenience was no longer significantly associated with vaccine acceptance in this group (*P* = 0.201). Worry about severe disease as well as protection for oneself were no longer significantly associated with vaccine acceptance in the above 50 years group (*P*-values = 0.421 and 0.920, respectively).

As the vaccination campaign in Finland had been underway for close to 4 months by the time of data collection, we added an additional question regarding vaccine uptake in this fifth and final round of the Finnish adaptation of the COSMO study. We asked participants about their reaction when/if they had already been offered the vaccine and their intended reaction once one would be offered to them if they had not yet received an offer. The majority of participants over the age of 64 (79%) had already either received the vaccine or booked an appointment to receive it, while other age groups were mostly still waiting for an offer (Fig. 2). Notably, in all age groups, participants had either already received the vaccine/booked an appointment or were planning on getting vaccinated as soon as they were offered the vaccine (Fig. 2). However, there was a group of participants that were preferring to wait to get vaccinated. This group was fairly small in the oldest age group (6%) but grew in younger age groups, with 29% of under-30-years-olds responding thus (Fig. 2). Overall, only 6% of those who had already received an offer responded that they would rather wait before receiving immunisation, while 24% of those who had not been invited yet reported they would wait before booking an appointment.

**Fig. 2.**
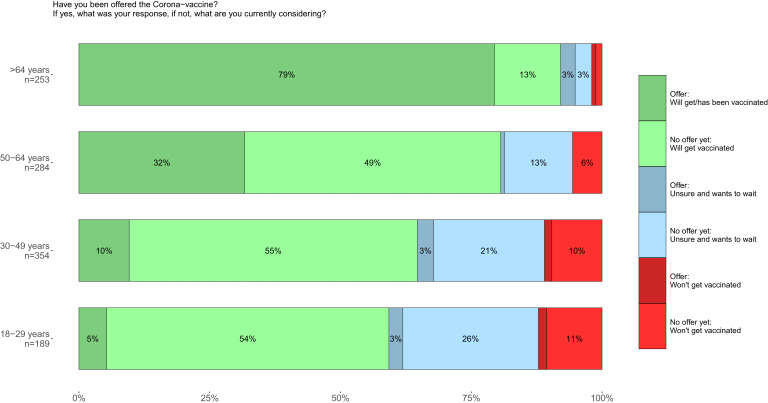
Self-declared response to a COVID vaccine offer during the fifth round (April 2021) by age group.

We have seen with the changes between November/December 2020 and April 2021 that acceptance of COVID-19 vaccines can change considerably within a few months and especially during a time with significant changes. Between November/December 2020 and April 2021, the question regarding vaccination turned from a hypothetical – if very possible in the near future – to a realistic scenario. In addition to knowledge of an increased risk associated with older age, this might explain differences in vaccine acceptance between age groups. At the time of data collection, vaccination had mostly only been offered to participants of older age. This is also reflected in the preference to wait among those who have already received an offer to get vaccinated *vs.* those who have not yet received an offer. However, it is not possible from our data to determine if this is dependent on age or on having received an offer as these two are highly intertwined. Additionally, the infection situation in Finland has shifted between the two time points with the country experiencing an increase in cases in late winter/early spring.

Overall, we are encouraged by the good uptake of the vaccine both in terms of intended uptake as well as real uptake shown in this latest round of data collection. We recognise the need to further engage younger generations to ensure good uptake and as concerns about their own protection and worry about severe disease remain significant predictors of vaccine acceptance in the younger group, it is important to emphasise that young people also have direct benefits from getting vaccinated. This needs to be kept particularly in mind for engagement with the group who would like to wait to get vaccinated. We currently know too little about this group and more research as well as engagement is needed to understand the motives and concerns of this particular group as well as of those who oppose vaccination.

## Footnotes

1 For methods, limitations, ethical clearance and general discussion, please refer to [1].
